# Plant-Based Polysaccharide Gums as Sustainable Bio-Polymers: Focus on Tragacanth Gum and Its Emerging Applications

**DOI:** 10.3390/polym17233163

**Published:** 2025-11-27

**Authors:** Shivani Dogra, Dhananjay Yadav, Bhupendra Koul, Muhammad Fazle Rabbee

**Affiliations:** 1 Department of Microbiology, School of Bioengineering and Biosciences, Lovely Professional University, Phagwara 144411, Punjab, India; shivani.41900129@lpu.in; 2 Center of Excellence in Aging and Brain Repair, Department of Neurosurgery, Brain and Spine, Morsani College of Medicine, University of South Florida, Tampa, FL 33612, USA; dhananjay11@usf.edu; 3 Department of Biotechnology, School of Bioengineering and Biosciences, Lovely Professional University, Phagwara 144411, Punjab, India; 4 Department of Biotechnology, Yeungnam University, Gyeongsan 38541, Gyeongsangbuk-do, Republic of Korea

**Keywords:** tragacanth gum, biocompatible, drug delivery, wound healing, nanocomposites

## Abstract

Plant-based natural polymers are gaining attention as ecofriendly alternatives to synthetic materials with applications in food, biomedical, pharmaceutical, and environmental science. Tragacanth gum (TG), a natural exudate obtained from *Astragalus* species, represents a unique polysaccharide with a complex molecular structure and distinctive rheological properties. It has been traditionally used for centuries as a stabilizer and emulsifier. Recent advances highlight its potential as a multifunctional biopolymer with industrial and biomedical potential. This review explores the structural characteristics, physicochemical properties, and modification strategies of TG, comparing it with other plant derived gums. Special emphasis is given to its applications in drug delivery, tissue engineering, wound healing, biodegradable packaging, and functional food formulation. Strengths such as biocompatibility and gel-forming ability but challenges remain including variability in quality, limited standardization, and issues with large scale production. Emerging trends, such as nanoformulations, hybrid polymer composites, and smart hydrogels, are also discussed. By positioning TG within the broader context of sustainable biomaterials, this review identifies key research gaps and proposes future directions to advance its role in the green polymer economy.

## 1. Introduction

Plant-derived polysaccharide gums have emerged as important candidates for replacing synthetic polymers due to their renewability, biodegradability, and structural versatility [1,2,3]. These natural polymers contribute significantly to the green bioeconomy by offering safe and sustainable alternatives across diverse sectors. *Astragalus gummifer* (Labill.) [4], a thorny perennial shrub of the family Fabaceae, is the botanical source of the natural polymer known as ‘Tragacanth Gum’, ‘Gond Katira’, or ‘Gum Katira’. Since ancient times, it has been incorporated into foods and pharmaceuticals as a stabilizer, emulsifier, and thickening agent [5,6,7,8]. In traditional systems of medicine, it has been employed to manage a wide range of conditions, including respiratory problems, gastrointestinal disorders, metabolic irregularities, and general weakness [1,9,10,11]. In recent years, research has highlighted its utility as a pharmaceutical excipient, given its biodegradable, biocompatible, non-toxic, and better thickening properties than acacia [12,13,14,15]. With the growing emphasis on plant-based therapeutics and sustainable biomaterials, *A. gummifer* has emerged as an important candidate that links traditional wisdom with contemporary biomedical and industrial applications [8,16,17,18,19].

The scientific basis of TG unusual functionality was established in mid-20th-century studies. Aspinall and Baillie (1963) [20] demonstrated that TG is composed of two distinct fractions: bassorin, an insoluble but highly swellable component, and tragacanthin, a soluble fraction rich in arabinogalactans. Earlier analyses, such as those by James and Smith (1945) [21], also revealed the presence of specific sugars, including fucose derivatives, helping to define its complex polysaccharide profile. Together, these foundational studies clarified the chemical framework behind TG’s swelling, water-binding, and gel-forming capabilities [6,22,23,24]. Although, early investigations provide the initial understanding of the rheological behavior and polysaccharide composition of gum derived from the Astragalus genus. Recent studies have substantially expanded this knowledge. Modern structural analysis using FTIR, NMR, and rheological methods have revealed detailed insights into swelling behavior, molecular architecture, and gelation dynamics of TG. A comprehensive review published in 2023 reports on its production methods, physicochemical properties, and various applications in biomedical, food, and industrial sectors [25,26]. Furthermore, a 2024 study demonstrates its potential as a multifunctional biopolymer for drug-delivery, scaffolding, and wound-healing systems. This underscores the correlation between molecular structure and application [27]. Collectively, these findings provide a refined and current perspective on structure–function relationships of TG.

Subsequent reviews and compositional studies in the 2000s expanded its relevance in food and pharmaceutical formulations, though most accounts remained descriptive rather than quantitative. For example, previous studies of gum exudates from the genus Astragalus reported the rheological characterizations and compositional analyses of multiple species [28]. Recent quantitative studies have begun to fill this gap, they reported detailed viscoelastic behavior, sugar profiles, and correlations between microstructure and functional performance. This provides a stronger mechanistic base for its biomedical and industrial applications [29,30]. In the past decade, however, TG has increasingly been investigated in advanced material science and sustainability contexts. Recent findings point to its potential as a biocompatible, renewable polymer for biomedical scaffolds, controlled drug delivery systems, wound dressings, functional food formulations, and biodegradable packaging [1,3,27,31,32,33,34]. These innovations highlight TG’s potential role in the “green polymer economy,” aligning directly with global sustainability initiatives aimed at reducing reliance on petroleum-based plastics [35].

**Figure 1:**
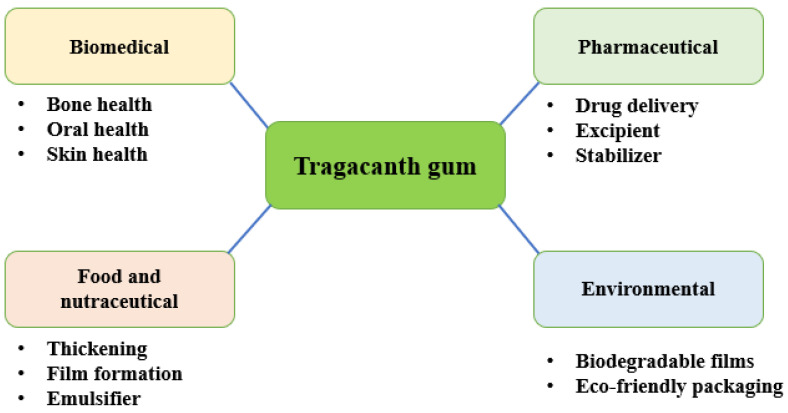
Overview of the major applications of Tragacanth gum across biomedical, pharmaceuticals, food/nutraceutical, and environmental sectors.

Current biomedical evidence not only validates these traditional uses but also expands its applications as a safe and effective excipient in drug formulations. Looking ahead, emphasis on novel drug delivery systems, mechanistic insights, and eco-friendly harvesting methods will further strengthen the role of *A. gummifer* as a valuable botanical resource in integrative medicine and green polymer science (Figure 1).

Despite the aforementioned advantages, several challenges persist. Comparative studies pertaining to the efficacy of other plant gums are limited, methods for standardized characterization are still lacking, and issues of industrial scalability remain unresolved. This review therefore aims to chart the evolution of TG research from its early chemical characterization to its modern applications while identifying critical research gaps and outlining future opportunities to fully harness TG as a sustainable biopolymer.

*A. gummifer* is native to Western Asia, particularly Iran, Iraq, Turkey, and Afghanistan, where it is cultivated and harvested on a large scale. The shrub typically grows in arid, dry, and mountainous regions at elevations of 1000–3000 m above sea level. It thrives in gravelly and rocky soils, demonstrating remarkable resilience in regions with low rainfall and extreme climatic conditions [36].

The plant is a small, spiny, perennial shrub that typically grows 30–60 cm in height. Its leaves are compound and pinnate, bearing numerous small, oblong leaflets. The stems are woody, gnarled, and often covered with short hairs, giving the plant a rough texture. The flowers are small, purplish to bluish in color, and usually arranged in clusters. A notable feature of this plant is its gum exudate, which is obtained by making incisions in the stems or bark; upon drying, the gum solidifies into ribbon-like flakes or tear-shaped pieces.

In Ayurveda, Unani, and Persian medicine, TG is described as a soothing, rejuvenating, and restorative agent. It is generally used for respiratory ailments (cough, sore throat, bronchitis), digestive disorders (constipation, colitis, burning sensation), and as a general tonic for debility and chronic fatigue [27,31,37,38]. TG shows a wide range of pharmacological activities that support its therapeutic potential. Its demulcent properties enable it to form a soothing layer over mucosal surfaces. This helps in relieving irritation associated with coughs and sore throats. Moreover, its polysaccharide-rich composition contributes to a variety of pharmacological effects, including demulcent, immunomodulatory, and anti-inflammatory effect, contributing to the reduction in systemic inflammation while promoting immune balance. The gum also possesses gastroprotective properties and offers relief in conditions such as ulcerative colitis and irritable bowel syndrome through its ability to calm and shield the intestinal lining. Additionally, it is recognized for its adaptogenic effects, as it improves vitality and helps to combat fatigue. Beyond these medicinal roles, it has gained importance as a pharmaceutical excipient, where its biocompatibility, emulsifying ability, and stabilizing properties make it highly suitable for use in modern drug delivery systems [39,40].

TG has a broad spectrum of application in both traditional and modern sectors. Traditionally, it is consumed after being soaked in water overnight. This serves as a gentle remedy for digestive discomforts and urinary problems. In the pharmaceutical field, its functional properties make it valuable as a binder, stabilizer, emulsifier, and controlled drug release agent in various drug formulations. The cosmetic industry also benefits from its natural thickening and soothing effects, incorporating it into creams, lotions, and gels. In the food sector, tragacanth is widely used as a natural thickener and emulsifier in beverages, sauces, and confectionery products. Even lower grade gum finds practical importance especially in textile processing and printing, reflecting its versatility, as a therapeutic and industrial resource [5,41,42].

## 2. Tragacanth Gum as a Sustainable Biopolymer

While numerous reviews discuss natural polymers, such as cellulose, starch, chitosan, and alginate, TG has received comparatively limited attention [43]. Its distinctive value lies in its highly branched molecular architecture, strong and stable emulsifying behavior, and relatively high resistance to microbial breakdown when compared with other plant gums. Positioning TG within the broader context of sustainability, green polymer development, and emerging biomedical applications provide a timely and relevant perspective in line with current research trends. While most published reviews emphasize the conventional roles of TG in thickening, emulsification, and stabilization, very few studies have investigated its broader potential as a sustainable biopolymer [32,33,43,44,45]. Earlier studies often overlook recent progress in modification strategies such as nanostructuring, crosslinking, and environmentally friendly processing methods, all of them can enhance its functionality [1,3,46]. Despite promising experimental evidence, its potential roles in biomedical engineering and environmental remediation have not been explored in depth. This review aims to fill these gaps by positioning TG within the broader concept of the circular bioeconomy, highlighting its opportunities in waste valorization, sustainable packaging, and advanced material innovations.

TG is a natural polymer that is biodegradable, non-toxic, non-carcinogenic, and environmentally safe causing minimal long-term ecological impact [33]. These properties have supported its use in sustainable packaging. For instance, it has been combined with apple pectin and pomace extracts to produce active films with antioxidant properties, suitable for preserving food products [47]. Similarly, TG-based films enriched with essential oils have shown enhanced antimicrobial activity, further highlighting their potential in eco-friendly packaging applications [48].

Despite this TG promise, more detailed life cycle assessments (LCAs) are necessary to fully evaluate its environmental benefits. Early studies indicate that replacing conventional plastics with TG-based materials containing packaging could reduce carbon footprints, but the challenges remain in industrial-scale production and processing require optimization to achieve these benefits effectively [49]. Life-cycle assessments of biopolymers suggest that replacing petroleum derived plastics with biopolymer-based composites can reduce carbon footprints.

## 3. Structural and Physicochemical Features of Tragacanth Gum

### Chemical Composition and Functional Properties

**Figure 2:**
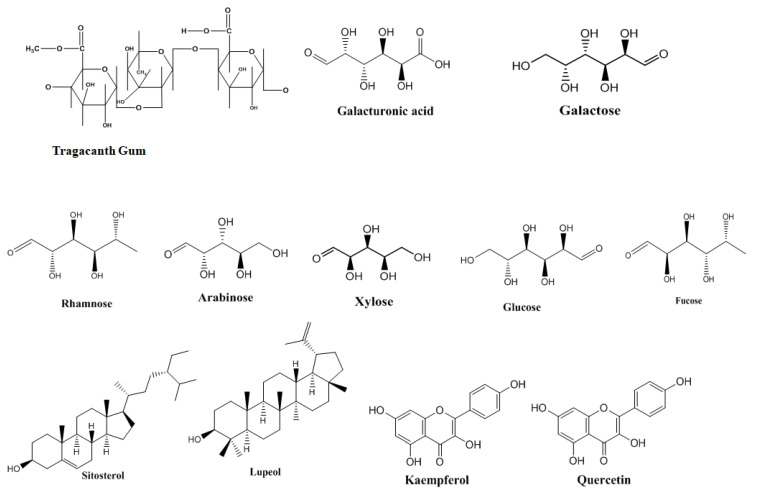
Chemical structures of TG and its composition.

TG is composed mainly of complex polysaccharides, with two principal fractions: bassorin (60–70%), which is insoluble in water but swells to form a gel, and tragacanthin (30–40%), which is water-soluble and yields a colloidal solution. In addition to these fractions, the gum contains various sugars such as D-galacturonic acid, D-galactose, L-fucose, L-arabinose, xylose, rhamnose, glucose, and other uronic acids [6]. It also contains proteins in the range of 2–4% and minerals, primarily calcium and magnesium salts, at about 1–2%. Trace amounts of phytochemicals, including β-sitosterol, lupeol, kaempferol, and quercetin are also present, contributing to its chemical complexity and biological significance. Figure 2 shows the intricate, highly branched polysaccharide structure of TG, mainly made up of galacturonic acid, galactose, arabinose, and other sugar units. This branched configuration is responsible for its distinctive flow properties, stable emulsifying ability, and resistance to microbial breakdown, setting it apart from other plant-based gums. The rheological behavior of TG varies between its two components. Bassorin generates thick, highly viscous, gel-like solutions even at low concentrations, making it highly effective thickening agent. In contrast, tragacanthin exhibits properties more like polymer system in semi dilute to concentrated solutions, providing controlled viscosity suitable for wide range of applications [33].

TG has a notable advantage ofremaining stable over a broad pH range (1–10) and under varying temperature conditions, makingit different from other plant-derived gums such as xanthan gum, guar gum, and gum Arabic, which are less resistant to environmental changes [31,50].

However, the performance and the composition of TG are not uniform. They can vary depending on the factors such as species of *Astragalus*, geographic origin, and extraction methods. So, the variations among exudates from different Iranian *Astragalus* species have been shown to influence both physicochemical and rheological characteristics, which directly affects their suitability for specific industrial or biomedical applications [51].

## 4. Advanced Materials Perspective

TG, a natural polysaccharide obtained from the exudates of *Astragalus* species, has emerged as a promising material in recent advanced materials research. Over the past two decades, its distinctive physicochemical characteristics such as strong water absorption capacity, inherent biocompatibility, and complete biodegradability have positioned it as a versatile candidate for innovative applications. TG has been widely investigated in the development of nanocomposites, functional hydrogels, and hybrid polymer systems, where these properties contribute to improved mechanical strength, environmental safety, and biomedical compatibility.

### 4.1. Nanocomposites: Enhancing Mechanical and Barrier Properties

The incorporation of TG into nanocomposite systems has been shown to markedly enhance functional properties such as mechanical strength, barrier performance, and antimicrobial activity. For example, TG gelatin films reinforced with zinc oxide nanoparticles (ZnO-NPs) demonstrated substantial improvements in tensile strength and Young’s modulus, while also exhibiting reduced water vapor permeability. These effects are largely attributed to the development of new chemical interactions and increased crystallinity within the polymeric matrix [52,53].

Beyond food and packaging applications, TG-based nanocomposites have also been explored for environmental remediation. A notable example is the synthesis of a ZnFe_2_O_4_/SiO_2_/TG nanocomposite, which showed high adsorption efficiency for methylene blue dye, underscoring its potential utility in wastewater treatment and related environmental applications.

### 4.2. Hydrogels: Applications in Drug Delivery and Tissue Engineering

TG-based hydrogels have been widely studied for applications in controlled drug delivery, wound healing, and tissue engineering [27,31,54,55]. Their high-water retention capacity, adjustable mechanical behavior, and ability to encapsulate therapeutic agents make them attractive platforms for biomedical use. In one approach, TG hydrogels were reinforced with bioactive ceramic particles to form hybrid scaffolds featuring controlled porosity and sustained growth factor release, which supported cartilage repair and regeneration. Similarly, TG has been combined with cellulose nanocrystals to design all-polysaccharide hydrogels, thereby avoiding the use of toxic crosslinking agents [56,57]. Using advanced fabrication methods such as 3D printing and freeze-drying, these hydrogels have been processed into porous scaffolds suitable for tissue engineering applications [58,59,60].

### 4.3. Hybrid Systems: Synergistic Enhancements in Material Properties

The incorporation of TG with other biopolymers, including chitosan, poly(vinyl alcohol) (PVA), and alginate, has enabled the creation of hybrid systems with enhanced functional properties, such as improved film strength, solubility, and thermal stability [60]. Chitosan/TG/PVA composite films incorporated with cinnamon essential oil nano emulsions demonstrated strong antimicrobial activity up to 95% inhibition against *E. coli* and *S. aureus* [61]. Similarly, TG with *Artemisia vestita* extract [62] promoted nearly 95% wound closure in vitro within 24 h, highlighting their potential for sustainable dressing and food packaging applications [8,63,64].

Beyond films, TG has also been employed in the fabrication of hybrid nanofibrous scaffolds for tissue engineering. Nanofibrous composites composed of chitosan and TG demonstrated favorable mechanical performance and biocompatibility, indicating their suitability for supporting tissue regeneration and other biomedical applications.

## 5. Biomedical Innovations

Over the last decade, TG has moved from a traditional pharmaceutical excipient into a multifunctional biomaterial with its diverse biomedical applications. In research, its popularity comes from its natural advantages that are biocompatibility, biodegradability, non-toxic degradation or by products, and its unique gel-forming ability have been central to this transition [35].

### 5.1. Wound-Healing Applications

One of the most primary focused areas is its wound healing applications. It has been incorporated into films, hydrogels, and nanofiber mats, often combined with plant extracts, silver nanoparticles, or antibiotics [65,66]. These formulations speed up tissue repair by keeping moisture retention and protect wound from microbes. In vitro scratch assays and small animal studies demonstrate enhanced wound closure rates and reduced bacterial load when compared with untreated control [67,68].

### 5.2. Drug-Delivery Systems

TG has been explored into the drug delivery system. Researchers have designed mucoadhesive carriers, pH-responsive hydrogels, and magnetic/thermo-responsive carriers [69]. These systems allowed for targeted and controlled drug release kinetics, particularly helpful for anticancer drugs, antibiotics, and bioactive molecules. For example, TG-based pH-sensitive hydrogels have shown targeted release in simulated gastrointestinal fluids conditions, highlighting their potential for oral drug delivery [27]. Similarly, TG magnetic composites have shown triggered responsive platform for cancer therapy, although validation remains limited to in vitro models or lab experiments [70].

### 5.3. Tissue Engineering and Regenerative Medicine

In the tissue engineering and regenerative medicine, TG has been blended with natural and synthetic polymers like chitosan, silk fibroin, collagen, and PVA to fabricate scaffolds. This will improve mechanical integrity, porosity, and cytocompatibility of scaffolds [71]. These scaffolds support cell adhesion and proliferation, making them promising for skin, bone, and cartilage regeneration [50]. Recently, in 3D bioprinting, TG has been utilized as a rheology enhancer in bioink. This is improving extrusion behavior and structural fidelity of printed constructs [72].

When compared with other natural gums such as xanthan gum, guar gum, and gum Arabic, TG shows strong film-forming ability, higher viscosity, stability, and more cell-friendly degradation. These qualities position it as a competitive alternative and attractive option for broader biomedical applications [50].

Despite these advances, the majority of research remains preclinical and laboratory based. Many studies are limited to in vitro cell assays or small animal wound models, leaving research gaps in toxicological profiling, long-term biocompatibility, and clinical validation. Future research must prioritize translational research, particularly well-structured animal experiments and early-phase clinical trials, to confirm its safety and efficacy in real-world biomedical applications. Table 1 compares TG with both synthetic and bio-based polymers from a green-economy perspective. The table outlines key aspects such as origin, biodegradability, renewability, safety, common applications, advantages, and limitations are highlighted, demonstrating the potential of TG as a sustainable and non-toxic substitute for petroleum-based polymers in packaging, biomedical, and coating applications.

**Table 1:** Comparative analysis of TG and synthetic/bio-based polymers from a green-economy perspective.

S. No.	Type of Polymer	Origin	Biodegradability %	Tensile Strength	O_2_ Barrier *	Cost *	Renewability	Toxicity Concerns	Applications	Green Economy Advantages	Limitations	Ref.
1.	TG (Natural polysaccharide)	*Astragalus* sap;	85–95%	15–30	1000–1500	8–15	Fully plant-based; minimal fossil-fuel use	Non-toxic; food-grade; pharma-safe when purified	Edible films, thickeners, and drug-delivery hydrogels	Renewable and low-toxicity	Hydrophilic and moisture-sensitive;	[27,73]
2.	Polyethylene (Synthetic, petroleum based) PE	Petrochemical; ethylene-derived	<1%	20–35	100–300	1–1.5	Non-renewable; fossil-fuel based	Generally inert; additives may be hazardous;	Packaging films, bags, and containers;	Low cost; strong mechanical and barrier properties	Fossil fuel dependence; environmental persistence; plastic pollution; GHG emission	[74]
3.	Polyethylene terephthalate (Synthetic) PET	Petrochemical; terephthalic acid + ethylene glycol	<5%	50–75	10–20	1.2–1.8	Non-renewable; bio-based PTA under research	Food-contact safe when processed correctly; microplastic and additive concerns	Bottles, fibers, packaging films	High strength, clarity, barrier properties; established recycling streams	Persistent if unrecycled; and limited replacement.	[75]
4.	Polylactic acid (PLA); bio-based, industrial polymer	Fermentation of sugars → lactic acid → polymerization; corn/sugarcane feedstocks	85–95%	50–70	500–800	2–3	Partly renewable; land use and fertilizer inputs matter	Low toxicity;	Compostable cups/packaging, fibers, 3D printing	Bio-based; good mechanical properties; familiar processing	Requires industrial composting; environmental fragmentation; agricultural footprint	[76,77]
5.	Polyhydroxyalkanoates (PHA); microbial biopolymer	Microbial fermentation of organic feedstocks, including waste streams	95–100%	20–40	200–500	6–8	Renewable; feedstock-flexible, can use waste streams	Low toxicity; biocompatible	Bioplastics, packaging, medical devices	Excellent biodegradability; strong green credentials; viable fossil–plastic replacement	Higher production cost; scale limitations; mechanical properties variable	[78,79]
6.	Starch-based polymers; natural polysaccharide	Plant starch (corn, potato, cassava)	90–100%	5–30	1500–2500	2–5	Renewable; abundant and low-cost	Low toxicity; food-grade	Disposable cutlery, films, fillers, blended bioplastics	Low-cost, renewable, compostable; blends with tragacanth improve film flexibility	Moisture sensitivity; lower mechanical strength; requires plasticizers or blending	[80]
7.	Cellulose/nanocellulose; natural polysaccharide	Plant cellulose (wood, pulp); nanocellulose	>95%	100–200	5–50	4–10	Renewable; wood and agricultural residues	Low toxicity; biocompatible	Reinforcements, films, packaging, composites	Strong mechanical properties; green profile; blends with tragacanth enhance composites	Requires controlled processing; energy and chemical inputs needed	[81]
8.	Chitosan; natural polysaccharide	Deacetylated chitin from crustacean shells or fungi	80–95%	25–50	800–1200	10–20	Renewable; waste seafood shells or fungal biomass	Generally biocompatible; allergy risk minimized through processing	Wound dressings, films, active packaging, coatings	Biodegradable; antimicrobial; complements tragacanth for biomedical or packaging uses	Higher cost; variability in quality; limited solubility at neutral pH unless modified	[82]

Note: Tensile strength (MPa); Oxygen barrier * (cm^3^·µm/m^2^·day·kPa), Cost *: (USD kg^−1^); GHG (Greenhouse Gas).

### 5.4. Material Properties and Biological Evaluation

The performance of TG in hydrogel applications depends strongly on its physical cross-linking behavior, chemical composition, and thermal mechanical stability [27]. TG contains both acidic and neutral polysaccharide fractions rich in arabinose, galacturonic acid, and fucose. These facilitate hydrogen bonding and ionic interactions in composite networks [83]. These interactions contribute to high water-retention capacity and adjustable gel strength characteristics. TG-based hydrogels show high swelling capacity up to 2000–2600%, elastic moduli ranging between 0.5 and 2 MPa, and thermal stability up to 250–300 °C before degradation [84]. Cross-linking with agents such as citric acid, borax, or poly (vinyl alcohol) has shown elasticity, tensile strength, and aging resistance. Various studies have shown that these hydrogels maintain their structural under repeated hydration drying cycles. These features support TG suitability for packaging and biomedical applications.

The branched polysaccharide structure of TG provides resilience during storage and heat exposure by limiting collapse of the network. Thermogravimetric analyses and differential scanning calorimetry (TGA/DSC) reveal multistage degradation behavior and glass-transition temperatures around 85–95 °C, consistent with other plant-derived biopolymers [85]. Composite materials based on TG integrated with plant-derived extracts or ZnO metallic nanoparticles have shown effective bacteriostatic activity. For instance, TG-ZnO composite hydrogels suppressed growth of *S*. *aureus* and *E*. *coli* and significantly accelerated burn-wound closure in animal models [86]. Likewise, TG/chitosan films and TG/Ag nanocomposites demonstrated broad-spectrum antibacterial effects attributed to synergistic ionic and polymeric mechanisms [64]. In terms of biocompatibility, in vitro studies using cell lines such as L929, HaCaT and NIH-3T3 have shown TG-based hydrogels to be non-toxic with cell viability typically exceeding 90–100%, and active fibroblast proliferation and migration were observed in vivo in rat and mouse wound-healing studies, with minimal inflammation [87]. Altogether these investigations show that TG hydrogels can be mechanically stable, thermally resilient, and biologically compatible materials suited for biomedical applications. However, most of the antibacterial and cytotoxicity data are restricted to laboratory and small-animal models; full toxicological profiling and clinical validation are still required for regulatory advancement.

## 6. Processing and Modification Techniques

TG has several inherent drawbacks including low solubility, inconsistent rheological behavior, and moderate mechanical strength. These limitations have driven significant research into processing and modification strategies [88]. Over the past decade, green chemistry approaches have gained particular attention, focusing environmentally sustainable methods to enhance the TG functionality for advanced applications.

### 6.1. Chemical Modifications Approaches

Chemical modifications, including carboxymethylation, acetylation, and periodate oxidation, have been widely applied to increase solubility, swelling behavior, and adjust biodegradation profiles [33]. Importantly, these derivatization strategies allow control over functional group density, making TG serve as a platform for applications like drug delivery systems and tissue engineering scaffolds.

### 6.2. Physical and Green Processing Techniques

Newer methods such as microwave-assisted graft polymerization and solvent-free crosslinking have emerged as faster and energy-efficient alternatives to conventional chemical methods. These approaches not only reduce solvent waste but also improve grafting efficiency, supporting the development of TG-based hydrogels and nanocomposites with stronger mechanical performance and higher thermal stability [89].

### 6.3. Enzymatic and Biological Functionalization

Enzymatic approaches represent another sustainable route using hydrolysis and selective cleavage to adjust molecular weight distribution and viscosity. This preserves the biocompatibility of TG while optimizing its performance in food formulations and pharmaceutical excipients.

### 6.4. Composite and Blending Strategies

Blending TG with other biodegradable polymers like PVA, polylactic acid (PLA), and chitosan have been shown to significantly enhance its film-forming capacity, tensile strength, and barrier properties. These blends broaden TG’s potential in areas like biodegradable packaging and wound dressings [65,66]. Similarly, the addition of nano fillers such as cellulose nanocrystals, graphene oxide, or metallic nanoparticles has improved interfacial bonding, dispersion uniformity, and functional responsiveness, enabling applications in smart materials and sensors [8].

Overall, these diverse modification techniques demonstrate TGs adaptability to chemical, physical, and biological interventions. However, challenges remain in scaling up green processes, ensuring consistency batch-to-batch, and conducting long-term safety assessments of nanocomposite formulations. Addressing these issues will be key to transitioning TG from experimental research to industrial and clinical applications.

### 6.5. Development of Tragacanth-Based Composites and Their Functional Roles

The use of TG in combination with other natural or synthetic polymers has become a significant approach to compensate for its low solubility and to enhance its mechanical and functional attributes. TG’s anionic character and multiple hydroxyl/carboxyl groups enable strong hydrogen-bonding and electrostatic interactions with biopolymers like chitosan, gelatin, alginate, PVA, and PEG [26]. Acting as a stabilizer and reinforcing component, TG improves the dispersion and uniformity of fillers or active particles within polymer blends and films. For example, TG/PVA and TG/chitosan nanocomposites show enhanced tensile strength, reduced brittleness, and greater moisture resistance due to intermolecular cross-linking [60]. When used as a coating or encapsulation matrix, TG also affords excellent emulsion stability useful for pharmaceutical and food-emulsion systems. In controlled-release systems, TG’s swelling behavior and pH responsiveness render it a valuable carrier. Hydrogels based on TG with alginate or gelatin have shown biphasic release profiles (an initial burst followed by slower diffusion), making them suitable for wound-healing or oral drug-delivery applications [55]. Embedding nanoparticles (e.g., silver, zinc oxide, silica) or therapeutic agents further add antimicrobial or combined delivery functionality. The performance of TG-based composites can be tuned by adjusting polymer ratios, cross-linker concentrations, and drying/post-processing conditions. This enables control over mechanical properties (e.g., Young’s modulus in the MPa range) and thermal stability (to ~250 °C) supporting their potential in biomedical scaffolds and packaging films [32]. Overall, TG-based composite systems represent a versatile, eco-friendly platform for stabilization, mechanical reinforcement, and controlled release, positioning TG as a key biopolymer in designing multifunctional materials across food, pharmaceutical, and biomedical fields.

## 7. Comparative Analysis with Other Plant Gums

TG is often studied alongside with other commonly used plant-derived gums such as xanthan gum, guar gum, and gum Arabic. This highlights both shared properties, functionalities, and distinctive advantages. TG maintains a stable viscosity across a wide pH range and under thermal stress, unlike guar gum which provides high viscosity but is sensitive to changes in pH. This resilience makes it particularly well suitable for food and pharmaceutical products subjected to diverse processing conditions [1,31].

TG exhibits stronger gel-forming capacity, which is a property linked to the combined action of its soluble fraction tragacanthin and insoluble fraction bassorin while compared with xanthan gum. These components contribute synergistically to rheological stability, swelling, and build robust gel networks. As a result, TG shows particular promise in applications such as wound healing materials, controlled drug release, and tissue engineering scaffolds where biocompatibility and durable structures of gels are essential [26,57].

Gum Arabic, in contrast, is primarily valued for its emulsifying efficiency and low viscosity. This makes it well suited to beverages, confectionery, and flavor encapsulation. However, TG surpasses gum Arabic when structural integrity, film formation, and rheological stability are required. These properties extend its potential use beyond the food industry into biomedical engineering, sustainable packaging, and 3D bioprinting [8,66].

Overall, TG emerges as a technically versatile yet underexplored biopolymer. While its multifunctionality and environmental stability offer clear benefits, current research is fragmented, with relatively few standardized comparisons among plant-derived gums. Future studies employing harmonized evaluation frameworks are needed to fully understand TG’s comparative advantages and industrial applications.

To better highlight the uniqueness of TG, a comparative summary of its key physicochemical and functional characteristics with other common plant gums such as guar gum and xanthan gum has been presented in Table 2. This analytical comparison clearly demonstrates the superior pH and temperature stability, stronger gel-forming capacity, and broader biocompatibility of TG compared with these counterparts.

The comparative properties presented in Table 2 possess that TG exhibits superior rheological and greater structural stability relative to other commercial gums. These attributes highlight its promise as a multifunctional and eco-friendly biopolymer. This aligns well with the principles of the prompting green polymer economy.

**Table 2:** Comparative properties of major plant-derived gums: tragacanth gum, xanthan gum, guar gum, and Gum Arabic.

Properties	Tragacanth Gum	Guar Gum	Xanthan Gum	Gum Arabic	References
Microbial/botanical source	*Astragalus* spp. Exudates *(A. gummifer, A. microcephalus)*	*Cyamopsis tetragonoloba* (seed endosperm)	*Xanthomonas campestris* (bacterial fermentation)	Exudate of *Acacia Senegal* and *A. seyal* trees	[90,91]
Main composition	Galacturonic acid, fucose, arabinogalactans and rhamnose	Galactomannan (Mannose: Galactose: 2:1)	Beta-D-glucose backbone with glucuronic acid and mannose side chains	Arabinogalactan with Ca, Mg, K salts of glucuronic acid	[33,90,91]
Solubility behavior	Partially soluble as tragacanthin soluble and bassorin swellable	Highly soluble in cold water	Completely soluble and forms pseudoplastic solutions	Highly soluble in cold water and generally forms low viscosity solutions	[92,93]
Rheology	High viscosity, stable at temperature variations and pH 1–10	Very high viscosity but pH sensitive	High viscosity, shear thinning and stable under moderate pH	Mild pseudoplasticity at high concentration	[92,93]
Gel forming ability	Strong due to tragacanthin and bassorin fractions	Moderate, limited gelation and forms viscous solutions	Viscosity control but poor gelation	Poor gel formation but primarily acts as emulsifier	[90,91]
pH and Thermal stability	Excellent pH and thermal tolerance (pH 2–10 and thermal stability: 180–220 °C)	Moderate tolerance and viscosity decrease with acids pH 5–8; thermal stability: 180–200 °C	Moderate to good. (pH 4–9; thermal stability: 200–230 °C	pH 3–9; thermal stability: 160–180 °C	[90,91]
Emulsifying capacity	Excellent emulsifying, stable emulsions and cohesive films	Good thickener and moderate emulsifying	Excellent stabilizer but limited film strength	High surface activity, film forming ability and excellent emulsifier	[90,91,92]
Biocompatibility	Biodegradable and non-toxic	Food grade and non-toxic	Generally safe but salt sensitivity limits its uses	Generally same, excellent in food and pharma	[33,90,91,92]

## 8. Applications of TG

**Figure 3:**
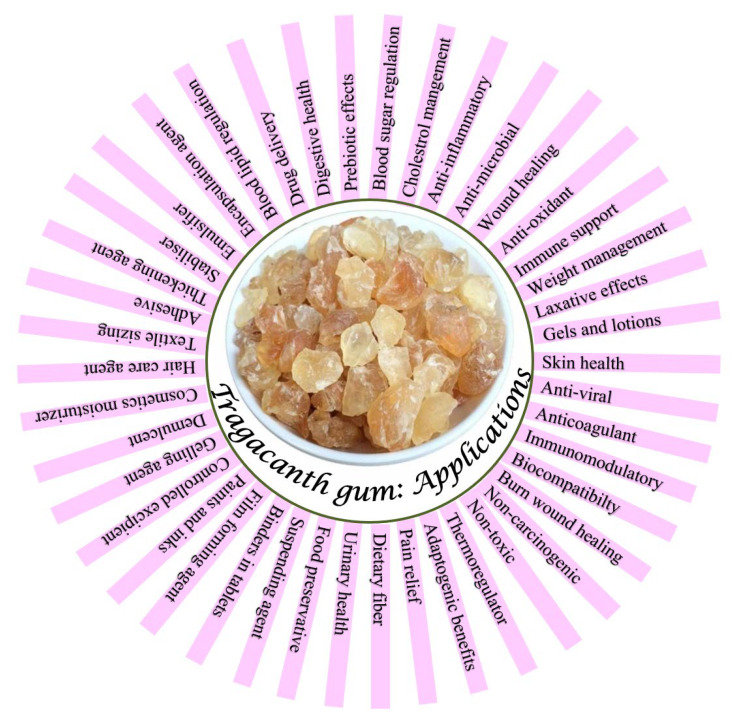
Visual representation of the wide-ranging applications of tragacanth gum, including therapeutic properties (antioxidant, anti-inflammatory, antiviral, anticoagulant), health benefits (digestive health, blood sugar regulation, immune modulation), cosmetic uses (skin and hair care), and functional applications, such as wound healing, biocompatibility, laxative effects, gels, lotions, and dietary fiber.

To date, no review has presented a comprehensive table that systematically compiles recent studies on TG-based biomedical hydrogels while directly comparing their methodologies, primary findings, and research gaps. The following section provides a detailed discussion of the diverse applications of TG, supported by a visual overview in Figure 3.

### 8.1. Biomedical Applications

In vivo investigations of biomaterials based on TG most often utilize rodent models, particularly rats and mice, due to the established protocols for assessing biocompatibility, toxicity, and wound healing in these species. For instance, full-thickness excisional wound models in rats are frequently employed when evaluating wound-healing and antimicrobial film applications of TG-based systems, as they closely replicate key aspects of human cutaneous repair. In bone-regeneration and tissue-engineering research, defect models in rats or rabbits permit the assessment of scaffold integration, new tissue formation, and functional recovery. In oral and drug-delivery applications, rat gastrointestinal models are commonly used to investigate absorption, release kinetics, and for the biocompatibility of TG-based hydrogels and films. Collectively, these preclinical models provide credible evidence supporting the translational potential of TG-based biomaterials.

Although the biomedical promise of TG encompassing hydrogels, wound-dressing constructs, scaffolds, and drug-delivery platforms has been reviewed in detail Section 5, this section offers a concise overview of recent advances and translational challenges. TG-based biomaterials are increasingly engineered with advanced functionalities such as self-healing behavior, stimuli-responsive drug release and integration into bioprinting workflows. New studies show that TG can form hybrid scaffolds reinforced by nanoparticles or combined with other polysaccharides, thereby improving mechanical strength, bioactivity, and antimicrobial performance. Nevertheless, despite promising laboratory results, the number of clinically validated systems remains limited, and regulatory approval is still minimal. To realize the full biomedical potential of TG, future work should emphasize scalable and green manufacturing, rigorous toxicological profiling and standardized in vivo evaluation protocols. Key recent developments, as well as existing research gaps, are summarized in Table 3.

Recent years have seen a surge of interest in TG as a multifunctional biopolymer for biomedical applications, particularly in the design of hydrogels, scaffolds, and drug delivery systems. A wide range of strategies from polymer blending to nanocomposite fabrication demonstrate TG’s adaptability, but translational challenges remain.

Self-healing hydrogels prepared by blending TG with poly (vinyl alcohol) and borax exhibit rapid recovery of structure after mechanical disruption and favorable water retention, making them suitable for wound dressings. However, issues such as sterilization methods and long-term in vivo performance require systematic evaluation [87]. Similarly, TG-based magnetic and thermo-responsive hydrogels incorporating nanoparticles enable stimuli-triggered drug release, yet biodistribution and clearance pathways remain insufficiently characterized [94,95,96]. Furthermore, TG-based hydrogel systems still need systematic refinement to achieve uniform and reliable biomedical outcomes. Critical design factors include the crosslinking density, which directly affects hydrogel porosity, mechanical integrity, and swelling characteristics; the drug loading efficiency, which governs therapeutic dosage and release kinetics; and the degradation rate, which should correspond to the intended timeline for drug delivery or tissue repair. Fine-tuning these parameters through precisely controlled synthesis and computational modeling strategies can significantly improve the stability, reproducibility, and translational potential of TG-based drug delivery platforms.

In tissue engineering, TG chitosan hybrid scaffolds have shown improved porosity, mechanical integrity, and cell adhesion compared to TG alone, highlighting their suitability for regenerative applications. Nonetheless, large-animal implantation and functional recovery studies are still lacking. Parallel studies incorporating antimicrobial plant extracts into TG hydrogels demonstrate accelerated wound closure and antibacterial effects in vitro, though in vivo validation and standardization of phytochemical content remain unmet needs [97]. Table 3 summarizes the latest developments from the last 5 years in the use of TG in areas such as drug delivery, tissue engineering, wound healing, and regenerative medicine, while also highlighting current limitations and knowledge gaps that need to be addressed for broader clinical and translational applications.

**Table 3:** Overview of recent progress in last five years TG-based biomedical applications and research gaps.

S. No.	Synthesis and Characterization	Evaluation Methods	Main Findings	Limitations	Application	Ref.
1.	CMGT synthesis, poly sodium acrylate crosslink, FTIR, XRD, NMR, SEM, TGA	Aceclofenac release, Higuchi, Korsmeyer–Peppas	CMGT- based hydrogel exhibited favorable rheological properties and facilitated drug release predominantly	Potential aspects, such as long-term mechanical stability, in vivo or in situ effectiveness, and issues related to scale-up and reproducibility	Controlled drug delivery	[98]
2.	TG hydrogel, *A. vestita* extract, viscosity, spreadability	In vitro drug release, scratch assay (HaCaT cells), antimicrobial testing.	The hydrogel demonstrated effective wound closure (~95% within 24 h), notable antimicrobial activity, and exhibited pH-responsive behavior	Need animal or in vivo models to assess long-term stability and safety	*A. vestita* leaf extract for wound healing, topical drug delivery	[62]
3.	Thermo-responsive magnetic hydrogel, TG, anticancer drug loading, magnetic and polymeric characterization	Temperature and magnetic responsiveness, drug release	The incorporation of magnetic components with TG enhanced control over drug release	Further investigation is needed to assess in vivo anticancer efficacy, systemic toxicity, and biodegradation	Targeted cancer therapy, controlled drug delivery	[95]
4.	Hydrogel: TG + collagen + polyurethane, varying gum concentrations, swelling, viscoelasticity, pH-dependent degradation	Antibacterial activity, cytotoxicity, and immune-modulatory testing	High swelling (>2600%), strong antibacterial, biocompatible, immune-modulatory properties	Need to evaluate long-term in vivo wound healing, mechanical stability under stress, and scalability	Wound dressing, tissue engineering, and synthesis of immuno-modulatory biomaterial	[54]
5.		Murine excisional wound model; planimetry, histology (AA + castor oil + TG formulation)	Enhanced wound healing (~87% compared to~70% in controls).	Clarifying the underlying mechanisms, such as identifying active polysaccharide fractions, comparison with standard treatments, and dosage optimization	Topical wound healing formulation, and skin repair	[55]
6.	Nanohydrogel: TG + cellulose + g-C_3_N_4_; lornoxicam loading; drug loading characterization	pH and temperature-dependent release, cell viability	Biocompatible, pH and temperature-responsive release, controlled drug delivery potential.	In vivo studies, stability and biodegradability, immune response, industrial-scale formulation	Controlled drug delivery, and smart nanohydrogel system	[35]
7.	Systematic literature review, TG composition, gelation behavior	Biomedical applications, and drug delivery, cell therapy	TG is under-exploited biomaterial, potential in drug delivery and cell therapy highlighted	Lack of raw material standardization, and absence of direct comparisons with other gums	Drug delivery, cell therapy, and biomedical biomaterial	[26]
8.	ZnO-tragacanth composite hydrogel, characterization	Murine burn wound model.	Accelerated burn wound healing, and enhanced tissue regeneration	Lack of mechanistic studies (immune modulation), systemic toxicity, and dose optimization	Burn wound healing, and tissue regeneration hydrogel	[86]
9.	TG–CNF films, free/encapsulated cumin oil (CEO/CNE), ± oxygen absorber (OA)	Applied to turkey burgers (food application outcomes)	Ternary system (TG–CNF + CNE + OA) most effective, lowest microbial counts, reduced spoilage (TVN, TBARS), minimal pH rise/cooking loss, shelf-life ≥20 days	Industrial scalability, cost–benefit, consumer acceptance, and validation in other foods remain unexplored.	Turkey burger packaging, food preservation	[99]
10.	TG-g-PAMPS hydrogel; microwave-assisted copolymerization; AgNP synthesis; FTIR, TGA, XRD, SEM, EDS, TEM; swelling, biodegradation	Antibacterial, diclofenac release, kinetic modeling	SN improved swelling and antibacterial activity, gels biodegradable, drug release non-Fickian, fitted Korsmeyer–Peppas model	Lack of in vivo validation, cytocompatibility/toxicity, SN concentration effect on mechanics/degradation, scale-up, biomedical application	Antibacterial hydrogel, and controlled drug delivery	[100]
11.	TG hydrogel + Ca^2+^ crosslink; silk fibroin + in situ Fe_3_O_4_; biocompatibility characterization	Hemolysis, cytotoxicity, hyperthermia (SAR)	Good dispersibility, biocompatible, low hemolysis, supports cell growth, SAR up to 41.2 W/g	The study is limited by unclear long-term stability and biodegradation, limited assessment of heating performance, and absence of tests in real physiological or targeted therapeutic conditions	Cancer hyperthermia therapy, biocompatible nanocomposite	[101]
12.	Double-layer hydrogel: TG + PVA + GrF + PANI; XRD, conductivity, contact angle, FESEM, tensile	MTT, biodegradation	PANI reduced crystallinity, enhanced conductivity (TP: 20,481×; TPG: 1804×), improved tensile strength (4.59×), good hydrophilicity (61.4°), uniform GrF, biocompatible (>90% viability), full biodegradation in 2 months	In vivo TENS validation, long-term stability, large-scale fabrication, optimize conductivity–biodegradability balance	TENS devices, conductive biodegradable hydrogel	[102]
13.	GT coating (0–2%) on bell peppers	Weight loss, firmness, biochemical traits, antioxidant enzymes, marketability	1% GT most effective: reduced weight loss (10.46% vs. 18.92%), maintained firmness, antioxidants, enzyme activity (SOD 97.02 U g^−1^, CAT 24.38 U g^−1^), improved antioxidant capacity (81.74%), marketability (~75% vs. 40%)	Scalability, consumer acceptance, cost analysis, combining GT with other natural additives for extended preservation across different fruits/vegetables	Bell pepper preservation, natural edible coating, shelf-life extension	[103]
14.	Alginate/aloe vera films with TG (2–14%), evaluated mechanical, barrier, optical, biodegradability properties.		TG improved strength and flexibility, tensile strength ↑ (max 67.64 N/mm^2^ at 12%), WVP/swelling/thickness ↑, solubility ↓, films darker with higher TG.	Optimization for packaging, impact on active compounds (antimicrobials/antioxidants), real-food storage testing	Edible films, food packaging, biodegradable composites	[104]
15.	Oxidized TG (up to 57%), characterized by FTIR, ^1^H NMR, SEM, TGA, DSC, rheology	Antibacterial activity, hemolysis, cell viability tested	Oxidation ↓ pore size (23 ± 0.8 µm), rheology, thermal stability; new functional groups (hemiacetal, NMR 5.38 ppm); gained antibacterial activity; biocompatible (≤1% hemolysis, >85% viability)	Structure–function relationship, long-term stability, in vivo performance, scalable OTG synthesis	Antibacterial biomaterial, biomedical applications, tissue engineering	[105]
16.	ST–GT hydrogels (varied ratios), PVA crosslink; FTIR, FESEM, TGA-DSC	Dye adsorption (MB, CR), kinetics, biodegradability	ST/GT 0.5:1.5 most efficient: MB 97.6% (pH 10), CR 93.7% (pH 2) in 90 min; adsorption pseudo-second-order; fully biodegradable in soil	Real wastewater testing, regeneration/reuse, large-scale production optimization	Biodegradable hydrogels for dye removal, wastewater treatment	[106]
17.	Films with cherry wine pomace extract	WVTR, OTR, opacity	Improved barrier properties: WVTR 7.96–10.95 g/m^2^·d, OTR 0.50–0.94 cm^3^/m^2^·d·0.1 MPa; increased opacity (~19%); high antioxidant potential; eco-friendly	Mechanical strength, real food shelf-life testing, scalability, cost-effectiveness	Eco-friendly bioactive food packaging, antioxidant films	[47]
18.	Eutectogels: TG bassorin + ChCl–malic acid; solubility, tensile, rheology, adhesion, texture	DXM transdermal permeability, anti-inflammatory (rat RA model)	DXM solubility ↑>1700×, gel stable up to 80 °C, strong mechanical and adhesive properties; 20% water: highest skin flux (332.7 mg/cm^2^·h); reduced paw swelling and TNF-α, normal knee histology	Long-term stability, large-scale production, human transdermal efficacy, testing other therapeutics	Transdermal DXM delivery, anti-inflammatory eutectogel	[107]
19.	TG hydrogel encapsulation: beetroot juice + basil	Nano-nutraceutical formation outcomes	Retained nutrients (iron, folic acid, vitamins C, niacin, chlorophyll, sugars); preserved TG encapsulation ability at nanoscale; no chemical additives	Bioavailability, gastrointestinal absorption, long-term stability, in vivo human efficacy	TG-based nano-nutraceuticals, functional foods, nutrient delivery	[108]
20.	TG + chitosan + MnFe_2_O_4_ hydrogels; hydrophilicity, biodegradation, adhesion	Biocompatibility (hemolysis, MTT), antibacterial, biofilm inhibition, tooth de/re-mineralization	Biocompatible, strong tooth adhesion, 100% *S. mutans* inhibition, high activity vs. *S*. *aureus and E. coli*, biofilm inhibition ≥85.94%, promoted remineralization, prevented demineralization	In vivo validation, long-term oral stability, scalability for dental use, impact on oral microbiome	Dental caries prevention, remineralization, antibacterial dental hydrogel	[109]
21.	3D scaffolds TG + PVP (DIW printing); printability, shear-thinning, SEM porosity, FTIR	Hydrophilicity, degradation, cell proliferation (WST-8), antibacterial	Good printability and shear-thinning, interconnected porous structure, hydrophilic, controlled degradation (168 h), sustained lawsone release, prolonged antibacterial activity (*E. coli*, *S. aureus*), supported cell attachment/proliferation	In vivo wound healing, long-term stability, mechanical optimization, combined bioactive molecules evaluation	3D wound healing scaffold, antibacterial, controlled drug delivery	[110]
22.	TG-based films and coatings; polymer blending, chemical properties	Food preservation applications	Hydrophilic, biodegradable, renewable, edible; composite films improve mechanical, barrier, functional properties; coatings extend shelf-life, maintain food quality	Large-scale industrial processing, cost-effectiveness, mechanical/barrier optimization, diverse food storage evaluation	Food packaging, edible coatings, shelf-life extension, functional food preservation	[66]
23.	TG extraction, hydrogel formulation; physicochemical characterization	Drug delivery and tissue engineering	Biocompatible, biodegradable, high water absorption, controlled drug release, effective for bone, skin, cartilage, periodontal regeneration	Limited in vivo studies, physiological stability, long-term biodegradability, immune response, scalable industrial formulation	Drug delivery, tissue engineering, regenerative medicine	[27]
24.	PVA/GT/CNC nanocomposite films, CNC from sugarcane bagasse (steam explosion + acid hydrolysis), solution casting, TEM, SEM, FTIR, mechanical andswelling tests	Cytotoxicity, antibacterial assays, betel leaf extract loading	Transparent, mechanically strong (elastic modulus 1526 MPa, tensile 80.39 MPa), stable 7 days, swelling ↓ with CNC, non-toxic (95% L929 viability), strong antibacterial activity (*S. aureus*, *P. aeruginosa*)	In vivo wound healing, long-term stability and biodegradability, CNC dispersion optimization, large-scale production feasibility	Wound dressing, antibacterial films, regenerative biomaterials	[110]
25.	Self-healing hydrogel: Tragacanth + PVA + borax, one-pot synthesis; characterized by rheology, self-healing tests	Cytotoxicity (L929), scratch assay, real-time PCR (TGFβ1, TGFβ2, VEGF-A)	Good mechanical strength, high self-healing, non-toxic (>100% viability), enhanced cell migration and scratch healing, increased growth factor expression, higher borax ↑ storage modulus	In vivo wound healing validation, long-term biodegradability andstability, large-scale production optimization, clinical application	Wound healing, tissue engineering, regenerative hydrogel	[87]
26.	Literature review: cellulose derivatives and nanocellulose, physicochemical properties, bio-ink design, cross-linking	3D bioprinting, cell–matrix interactions	Biocompatible, biodegradable, cost-effective, printable; cross-linking tunes mechanics; functional groups/surface charge influence cell adhesion, survival, proliferation	Limited tissue-specific bio-inks, long-term stability, in vivo performance, optimized multi-component formulations	3D bioprinting, tissue engineering, bio-ink development	[111]

±oxygen absorber (OA)—with or without oxygen absorber; TEM—Transmission Electron Microscopy; SEM—Scanning Electron Microscopy; FTIR—Fourier Transform Infrared Spectroscopy, PCR—Polymerase Chain Reaction; TGFβ1—Transforming Growth Factor Beta-1, TGFβ2—Transforming Growth Factor Beta-2, and VEGF-A—Vascular Endothelial Growth Factor-A. CNF: Cellulose nanofiber; CEO: Cumin essential oil; CNE: Encapsulated cumin oil; OA: Oxygen absorber; PVA: Polyvinyl alcohol; GrF: Carboxylated grapheme; PANI: Polyaniline; GT: Gum tragacanth; ST: Starch; MB: Methylene blue; CR: Congo red; DXM: Dexamethasone; PVP: Polyvinylpyrrolidone; DIW: Direct ink writing; ↑—Increase; ↓—Decrease.

Mucoadhesive drug delivery formulations of TG developed as films or hydrogels have displayed strong adhesion to mucosal surfaces and predictable release kinetics for model drugs, but clinical pharmacokinetic studies are scarce [112,113]. At the nanoscale, TG-based nanohydrogels combined with cellulose derivatives or g-C_3_N_4_ exhibit tunable release under pH or temperature changes and enhanced mechanical stability. These systems, however, require further biodegradation and immunological profiling [114].

Metal polymer nanocomposites such as TG-silver and TG-ZnO show strong antibacterial activity and accelerated wound healing in small animal models, yet concerns about metal ion leaching, cytotoxicity, and environmental impact persist [115,116]. Bioadhesive formulations using TG for dental and soft-tissue repair have demonstrated promising bonding strength and cytocompatibility, but durability in the oral environment and controlled clinical evaluation are critical next steps [117].

**Figure 4:**
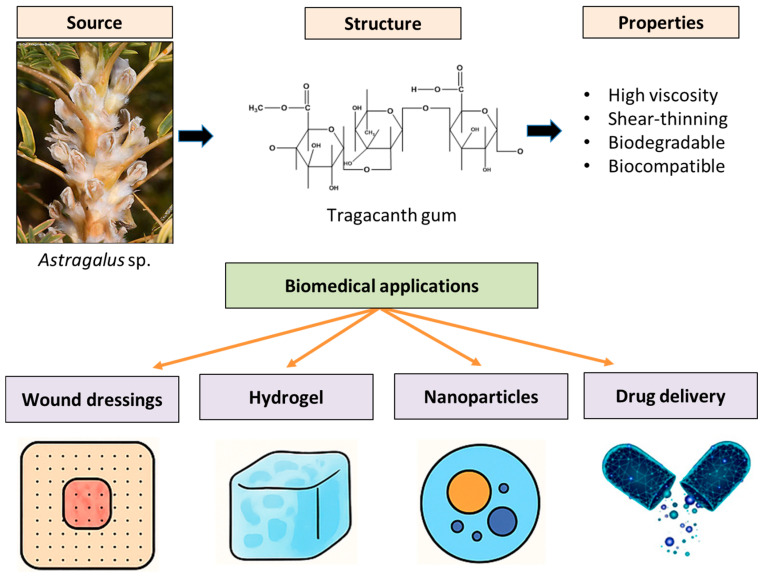
Overview of Tragacanth gum and its biomedical applications.

In the nutraceutical domain, TG has been employed for microencapsulation of probiotics and sensitive bioactives, extending shelf-life and enhancing survival during simulated gastrointestinal transit. Nevertheless, human bioavailability data and regulatory clarity remain limited [68]. Recently, TG has been incorporated into eutectogel systems using deep eutectic solvents, enabling us to improve the solubility of poorly water-soluble drugs and allow for sustained release behavior. While these findings are encouraging, the toxicological assessment of eutectic components and their long-term stability still remain a critical challenge [118]. Collectively, these studies affirm TG’s potential as a versatile biopolymer across biomedical applications as represented in Figure 4, but also reveal persistent gaps in its standardization, translational research, and regulatory readiness.

### 8.2. Food Industry

TG is an eye-catching sustainable biopolymer for food packaging and preservation as it is renewable, biodegradable, and has the ability to forming strong flexible films. When it is blended with other biopolymers such as alginate, pectin, or chitosan, TG improves the mechanical strength and barrier properties of edible coatings and active packaging films.

Incorporation of the plant extracts and essential oil provides additional antimicrobial and antioxidant effects. This helps to effectively extend the freshness and shelf life of perishable foods. Moreover TG-cellulose nanocomposite films further improve the tensile strength and reduce moisture and oxygen permeability, which reinforce their value for biodegradable packaging. However, key challenges remain for large scale production, cost optimization, and safety evaluation of active compounds migration. Overcoming these issues will support TG’s adoption as a viable and eco-friendly alternative to petroleum-based packaging materials.

TG has recently gained interest as a functional biopolymer for food preservation and sustainable packaging, due to its biodegradability, film-forming properties, and ability to carry active constituents. TG-based edible coatings on fresh fruits and vegetables have been widely tested in postharvest studies. These coatings effectively reduce water loss, slow down oxidative browning, and preserve nutritional quality, thereby extending shelf life. Despite such promising results, consumer sensory acceptance and adaptation to commercial-scale pack house operations remain underexplored [66,119].

Blended films of TG with pectin or alginate, often loaded with natural antioxidants and antimicrobials, show improved barrier properties and can act as active packaging by inhibiting microbial growth in perishable foods. However, data on the migration of bioactive compounds into real food matrices and regulatory clearance are limited [48,120].

In nanocomposite systems, TG films reinforced with cellulose nanocrystals (CNC) have shown substantial improvements in tensile strength and water/oxygen barrier properties, enhancing their potential for high-performance packaging. Nevertheless, extrusion-based trials at industrial scale are lacking [121]. TG has also been applied in delivery systems for lipophilic bioactive compounds like curcumin and carotenoids. Encapsulation and emulsification studies suggest improved stability during storage and gastrointestinal digestion, leading to controlled release. Yet, clinical validation of enhanced bioavailability in humans is still missing [122,123]. More recently, TG–chitin films incorporated with essential oils demonstrated antimicrobial efficacy against common food borne pathogens while retaining good mechanical performance. The challenge ahead lies in scaling production and verifying compostability under real industrial conditions [124].

TG-based composites with starch or poly(vinyl alcohol) have also been explored for biodegradable trays and single-use items. These lab-scale trials indicate that TG can replace certain petroleum-based plastics, though cost-effectiveness, extrusion, and molding performance in industrial contexts require further work [125]. Overall, TG’s versatility across coatings, films, and composites highlights its promise as a sustainable food-packaging material. Still, sensory acceptance, regulatory validation, and industrial scalability represent critical gaps for its commercial adoption.

### 8.3. Environmental and Water Treatment

The versatility of TG extends beyond food and biomedical domains, with recent studies demonstrating its potential in environmental sustainability and water purification. TG–starch hydrogels have been investigated as low-cost sorbents for dye removal from wastewater. Batch adsorption studies, fitted to isotherm and kinetic models, reveal that TG improves water retention and dye-binding efficiency, while offering biodegradability. Nevertheless, most findings stem from synthetic dye solutions; evaluation in industrial effluents with complex matrices and long-term regeneration stability remains limited [126].

Functionalized TG-based biofilters and sorbents have also been developed for heavy metal remediation. Bead formulations and chemical modifications enhanced adsorption of toxic cations such as Pb^2+^ and Cd^2+^. While initial results are promising, challenges persist regarding performance under competitive ion conditions and the absence of pilot-scale field trials. The biodegradability of TG-containing films has been validated in laboratory studies using soil burial and enzymatic degradation assays. These results confirm the gum’s compatibility with composting systems and its potential to reduce plastic waste. Yet, studies seldom examine gaseous emissions or degradation byproducts during composting, which are critical for environmental safety assessments [127].

More recently, TG has been explored as a matrix for supported photocatalysts such as graphitic carbon nitride (g-C_3_N_4_) and titanium dioxide (TiO_2_). TG improves nanoparticle dispersion and mechanical stability, enabling efficient degradation of organic pollutants under irradiation. However, the reusability and photostability of such composites under natural sunlight require further exploration before scale-up [128].

Collectively, these findings highlight TG’s promise as a sustainable biopolymer for environmental remediation. Addressing gaps related to byproducts degradation, field performance, and large-scale implementation will be crucial for its transition from laboratory innovation to the real-world applications.

Despite significant advancements in the application of TG for biomedical, food, and packaging applications, several limitations have been revealed in existing methodologies. Many studies relied mainly on descriptive observations without standardized testing procedures, which makes it typical to compare the results between different formulations [33,73]. Variations in the gum source, purification techniques, and crosslinking conditions have led to inconsistent rheological and mechanical performance. In some cases, TG-based films and hydrogels have demonstrated uncontrolled degradation, weak structural integrity, or poor bioactivity. This is due to inadequate optimization of polymer ratios and processing conditions. These failed or inconsistent formulations underline the need for systematic design strategies that are supported by quantitative data such as swelling index, tensile strength, and release kinetics.

Additionally, comparative studies between TG and other plant-derived gums like xanthan gum, guar gum, and gum Arabic often lack of clear performance benchmarks and uniform evaluation criteria [129]. While TG offers superior pH stability and gel-forming ability, the discrepancies remain regarding its viscosity behavior and long-term performance under industrial conditions [91]. Future research work should therefore adopt standardized characterization procedures and include mechanistic evaluation protocols to clarify why TG formulations achieve desirable functional outcomes while others do not [130].

## 9. Research Gaps and Future Perspectives

Most existing reviews on TG primarily highlight its traditional roles as a thickener, emulsifier, and stabilizer. However, its potential as a sustainable polymer for advanced material applications has received relatively little attention. In particular, recent modification approaches such as nano structuring, cross linking, and eco-friendly processing are seldom explored in depth. Similarly, discussions of its applications in emerging fields like biomedical engineering and environmental remediation remain limited. Moreover, forward-looking perspectives that connect TG to the circular bioeconomy, including waste valorization and sustainable packaging solutions, are largely absent from the literature.

### 9.1. Reviews, Overviews, and Comparative Analyses

Research on TGhas been extensively documented through narrative, systematic, and comparative reviews spanning its chemistry, traditional uses, and emerging applications. Narrative reviews published between the 2000s and 2021 emphasized the gum’s unique dual structure tragacanthin (a soluble arabinogalactan-rich fraction) and bassorin (an insoluble but highly swellable fraction) as the molecular basis for its rheological versatility. These properties explain TG’s longstanding role as a stabilizer and thickening agent in food and pharmaceutical systems. However, most early reviews were descriptive, lacking standardized methodologies or quantitative synthesis, which limits reproducibility and cross-study comparisons [26].

Systematic reviews conducted more recently have focused on TG’s biomedical potential, particularly hydrogels, films, and scaffolds for wound healing and drug delivery. Findings consistently indicate biocompatibility, tunable swelling, and controlled-release behaviors. Nevertheless, the literature remains dominated by in vitro and small-animal studies, highlighting the need for robust preclinical and clinical validation before translation to clinical use [27].

In the area of food packaging, reviews evaluated TG in edible films and coatings, often in combination with polymers such as alginate, chitosan, pectin, or cellulose nanocrystals. These formulations enhance barrier properties, biodegradability, and enable active packaging through antioxidant or antimicrobial incorporation. Despite encouraging outcomes, critical industrial data including migration testing, life-cycle assessments, and pilot-scale production remain under-reported [66].

Comparative reviews positioned TG against other plant gums (e.g., xanthan, guar, gum Arabic). While TG often demonstrated superior gelation capacity and pH tolerance. Gaps persist due to the scarcity of standardized head-to-head experiments under identical conditions, limiting direct industrial applicability [31,50].

In the fast-developing bioprinting field, TG has been identified as a promising additive to bioinks, improving shear-thinning behavior, and print fidelity. Yet, sterility protocols, optimization for cell viability during extrusion, and in vivo scaffold performance remain poorly explored [57]. Regulatory and sustainability-oriented reviews acknowledge TG’s promise as a renewable material but emphasize its underrepresentation in circular economy analyses. Standardized specifications, supply-chain transparency, and detailed environmental assessments are lacking, with no clear regulatory frameworks for food-contact or biomedical applications [26,72].

Finally, technological prospectuses have explored TG’s compatibility with nanofillers and role in smart composite systems, such as conductive hydrogels or magneto-responsive matrices. While these findings illustrate TG’s potential in advanced materials, unanswered questions remain regarding nanofiller toxicity, environmental persistence, and lifecycle impacts [8].

### 9.2. Processing, Functionalization, and Material Science

In the last decade, studies have made tremendous progress in tailoring the TG functional and structural properties. TG is driving its transition from a traditional thickener to a versatile material platform through various physical, chemical, and composite-based methods. Chemical modification approaches, such as periodate oxidation and carboxymethylation, have been proven to effectively adjust its solubility and degradation profiles, and enhance its ability to form crosslinked networks. These modifications expand its suitability for packaging and biomedical applications. Though determining the optimal degree of modification to balance improved its functionality with cytotoxicity risk remains an unresolved challenge [131]. Green processing strategies, including eco-friendly crosslinking and microwave-assisted graft polymerization, have been developed as energy-efficient alternatives to conventional chemical treatments. These approaches reduce chemical waste and processing time, offering more sustainable routes to functional TG derivatives. However, reproducibility at industrial scale and robust energy efficiency analyses are largely missing.

Emerging interest in TG-based conductive and composite hydrogels has highlighted its potential in wearable electronics and biomedical sensors. Incorporation of graphene and other conductive fillers improves flexibility and electrical responsiveness, making such composites promising for smart wound dressings. Yet, long-term cytotoxicity and biocompatibility of conductive fillers must be systematically assessed before clinical adoption [132]. In 3D bioprinting, TG has demonstrated value as a rheology modifier in bioink formulations. Blended with alginate or gelatin, TG enhances extrusion behavior, shear-thinning, and scaffold fidelity, thereby enabling more precise constructs. Still, key translational barriers remain, particularly the development of sterile, cell-laden printing protocols and validation of in vivo implantation outcomes [27].

Blending TG with synthetic polymers such as poly(vinyl alcohol) (PVA) or polylactic acid (PLA) has been reported to enhance tensile strength, thermal stability, and film durability. However, these advantages often come at the expense of biodegradability, raising questions about regulatory classification and environmental trade-offs [133,134].

Finally, advances in nanofiller dispersion and interfacial chemistry have improved homogeneity, barrier performance, and reinforcement in TG-based matrices. Strategies such as surface modification of cellulose nanofibers (CNFs), cellulose nanocrystals (CNCs), and graphene oxide have proven effective, though challenges related to interfacial aging, moisture sensitivity, and long-term stability remain [93,135].

Together, these studies underscore the adaptability of TG as a multifunctional biopolymer platform, while emphasizing the need for scalable, safe, and environmentally sound processing methods to unlock its industrial potential.

### 9.3. Standardization, Quality Control, and Supply-Chain Considerations

The quality and functional performance of TG (TG) are strongly influenced by its botanical origin and processing methods [136]. Comparative compositional studies consistently highlight marked variability in sugar composition, intrinsic viscosity, and molecular weight distribution across species and harvesting regions. Such heterogeneity directly impacts rheological behavior, solubility, and biofunctional properties, yet no internationally harmonized pharmacopeial or industrial standards exist to ensure consistent quality of TG in food, pharmaceutical, or biomedical use [137,138]. The absence of standardized quality parameters remains a critical barrier to its wider industrial adoption.

Parallel advances in pilot-scale extraction and drying optimization have demonstrated that adjusting drying kinetics, extraction solvents, and purification steps can significantly improve TG yield and reduce impurities. These technological refinements hold promise for industrial upscaling. However, comprehensive techno-economic evaluations and assessments of environmental sustainability are still limited [139]. Integration of life cycle analyses and standardized supply-chain audits will be essential to establish TG as a reliable, scalable, and sustainable biopolymer in global markets.

### 9.4. Scalability and Industrial Limitations

Despite TG promising multifunctionality, large-scale utilization remains constrained by several practical factors [33]. The yield and quality are highly dependent on Astragalus species, climate, and harvesting practices. This leads to inconsistency in composition and rheological performance [27]. Downstream processing is also challenged by extraction and purification steps, which increase the cost of production compared with starch-based and synthetic polymers. The absence of internationally harmonized specifications also complicates quality assurance and bulk procurement. In addition, limited availability of raw material and geographically concentrated mainly in arid regions of Turkey, Iran, and Afghanistan restricts continuous industrial deployment [89]. To achieve commercial scale up, future efforts should prioritize improved cultivation and extraction methods, establishing global quality standards, conducting techno-economic and life-cycle assessments which can evaluate cost–benefit and environmental impacts at industrial scale.

### 9.5. Clinical Status and Translational Prospects

Although multiple in vitro and in vivo (animal) studies have demonstrated the biocompatibility and wound-healing potential of TG in composite hydrogels, burn-wound models and controlled-release systems, no registered human clinical trials evaluating TG as an active biomaterial have yet been reported [27,33].

The existing evidence derived from laboratory or small-animal studies on TG-based wound dressings, hydrogels, and drug-delivery systems. They constantly reported accelerated wound closure, favorable cell viability, and controlled drug delivery [140].

The absence of clinical validation therefore highlights a key translational barrier. Before TG-based formulations approval for therapeutic use, comprehensive preclinical toxicology, pharmacokinetic analyses, and Phase I/II trials are required. This confirms their biodegradation behavior, safety, and therapeutic effectiveness in humans [26]. In addition, establishing standardized procedures for extraction, purification, and sterilization will be essential to ensure batch-to-batch reproducibility and regulatory compliance [27].

Going forward, collaborations among academia, pharmaceutical industries, and clinical research centers could facilitate the design of ethically approved clinical studies investigating TG in oral mucoadhesive films, topical wound-healing products, or biodegradable implants (Nawaz). Such trials will be crucial to convert TG’s laboratory-based promise into clinically accepted biomaterials for medical and pharmaceutical applications.

## 10. Summary and Conclusions

Research on TG has evolved from early studies on its chemical structure and rheology to contemporary explorations spanning biomedical, food, material, and environmental domains. Initial investigations demonstrated that TG’s composition and viscosity vary considerably with botanical source and geographic origin, highlighting the need for standardization and quality assurance in raw material utilization. In biomedicine, TG-based hydrogels are increasingly explored for drug delivery and wound healing. Formulations incorporating plant extracts, nanoparticles, or responsive polymers have shown antimicrobial properties, pH-sensitive release, and accelerated wound closure in vitro. Emerging stimuli-responsive systems, including thermo- and magnetically activated hydrogels, suggest potential in targeted anticancer therapy, although most remain at proof-of-concept stages with limited in vivo validation. From a materials perspective, TG has been combined with polymers such as poly(vinyl alcohol), cellulose nanocrystals, silk fibroin, and starch to create nanocomposites and scaffolds with enhanced strength, porosity, and cytocompatibility. These advances support applications in regenerative medicine, 3D printing, and bioink development, though studies on long-term biodegradability and biosafety remain sparse. In the food and packaging sector, TG has been employed in edible coatings and active packaging films often blended with pectin, alginate, or chitin to improve barrier performance, antioxidant activity, and shelf-life extension of fresh produce. While promising as a biodegradable plastic alternative, industrial-scale validation, and regulatory compliance require further attention. Environmental applications include the design of TG-based hydrogels and adsorbents for removing heavy metals and dyes from wastewater. Enhanced efficiency has been reported when TG is integrated with starch or nanomaterials, though real-world testing and regeneration performance are yet to be fully addressed.

TG stands out among other natural polysaccharides like guar gum, xanthan gum, and gum Arabic. Its dual structural components tragacanthin and bassorin impart pH and thermal stability, superior gel-forming ability, and rheological resilience across various environments. These properties make TG highly promising particularly for biomedical uses such as wound dressings, hydrogels, and biodegradable films where strength, flexibility, and biocompatibility are essential. Furthermore, its non-toxic and renewable nature aligns well with the objectives of sustainable material development. However, at the same time TG still faces challenges while compared with other gums. It includes variations in the composition depending on plant species, limited solubility, and difficulties in large-scale standardization and production. Future research should focus on establishing uniform standardized quality specifications, improving extraction efficiency, and processing methods. To expand its usability, ecofriendly techniques should be developed to enhance its functionality and industrial applicability. By addressing these challenges, TG can evolve from a traditional thickening agent into a benchmark biomaterial for the next-generation green polymer technologies.

## Data Availability

No new data were created or analyzed in this study. Data sharing is not applicable to this article.

## Abbreviations

The following abbreviations are used in this manuscript:

TG	Tragacanth Gum
TG–CNF	Tragacanth Gum–Cellulose Nanofiber
CNF	Cellulose Nanofiber
CNC	Cellulose Nanocrystals
CEO	Cumin Essential Oil
CNE	Encapsulated Cumin Essential Oil
OA	Oxygen Absorber
PVA	Polyvinyl Alcohol
GrF	Carboxylated Graphene
PANI	Polyaniline
ST	Starch
MB	Methylene Blue
CR	Congo Red
DXM	Dexamethasone
MNP	Magnetic Nanoparticles
PVP	Polyvinylpyrrolidone
DIW	Direct Ink Writing
RTC	Ready to Cook
FTIR	Fourier Transform Infrared Spectroscopy
TEM	Transmission Electron Microscopy
SEM	Scanning Electron Microscopy
FESEM	Field Emission Scanning Electron Microscopy
XRD	X-ray Diffraction
TGA	Thermogravimetric Analysis
DSC	Differential Scanning Calorimetry
NMR	Nuclear Magnetic Resonance
PCR	Polymerase Chain Reaction
TGFβ1	Transforming Growth Factor Beta-1
TGFβ2	Transforming Growth Factor Beta-2
VEGF-A	Vascular Endothelial Growth Factor-A
SAR	Specific Absorption Rate
SOD	Superoxide Dismutase
CAT	Catalase
POD	Peroxidase
WVTR	Water Vapor Transmission Rate
OTR	Oxygen Transmission Rate
